# Is outcome observed in performance of clinical tests associated with patient-reported outcome measures after patellofemoral pain treatment? Secondary analysis of data from a randomized clinical trial

**DOI:** 10.1016/j.bjpt.2026.101593

**Published:** 2026-06-24

**Authors:** Eliane de Morais Machado, Mark Matthews, Michael Skovdal Rathleff, Fábio Viadanna Serrão, Bill Vicenzino

**Affiliations:** a Department of Physical Therapy, Federal University of São Carlos (UFSCar), São Carlos, Brazil; b University of Queensland School of Health and Rehabilitation Sciences: Physiotherapy, The University of Queensland, QLD, Australia; c Ulster University, School of Sport and Exercise Science, Northern Ireland, United Kingdom; d Department of Health Science and Technology, Aalborg University, Denmark; e Department of physiotherapy and occupational therapy, Aalborg University Hospital, Denmark

**Keywords:** Anterior knee pain, Clinical assessment, Function, Rehabilitation

## Abstract

**Background:**

Clinical functional tests for patellofemoral pain (PFP), such as step-up, step-down and squat, are commonly used in clinic practice, although the extent to which changes in these tests reflect changes in patient-reported outcome measures (PROMs) remains unclear.

**Objective:**

Investigate how changes in step-up, step-down, and squat tests are associated with changes in PROMs in individuals with PFP.

**Methods:**

This secondary analysis used data from a randomized controlled trial (*N* = 218) comparing 12-week outcomes following either foot orthoses or 4-week hip exercise program. The number of pain-free repetitions in the functional tests of step-up, step-down and squat was assessed. PROMs included the Kujala, the Knee Injury and Osteoarthritis Outcome Scale (KOOS) subscales (Symptoms, Pain, Activities of Daily Living, Sporting and Recreation and Quality of Life (QoL), and the Global Rating of Change (GROC). Associations between change scores (differences between follow-up and baseline) in pain-free repetitions in the clinical functional tests and PROMs were analyzed using multivariate regression and binary logistic regression for dichotomized GROC (*p* < 0.05).

**Results:**

Data was available for 157 participants. There was a positive association between change scores in functional tests and PROMs, with explained variances ranging from 11 to 30%. The step-up test was weakly to moderately associated with KOOS Symptoms, Pain, QoL and Sport/Recreation. Step-down was associated with Kujala and KOOS Sport/Recreation. The squat test was not associated with any PROM.

**Conclusions:**

Improvement in step-up test is associated with better PROMs. Clinicians might use the step-up test to monitor progress in individuals with PFP.

## Highlights

• Pain threshold step-up test is most closely related to PFP PROMs.
• Squat test was not associated with any PROM investigated.
• Step-up test could compliment assessment of pain, quality of life and sport.
• Changes in step-up test in the clinic can be used to monitor treatment progress.

## Introduction

Patellofemoral pain is one of the most common musculoskeletal disorders and affects one in four youth and adults.^1^ The prognosis is not always favourable with over 50 % of people reporting pain up to 8 years after diagnosis.^2^ Patellofemoral pain negatively impacts daily activities, physical activities and causes withdrawal from sports participation and overall quality of life.^3^

Patellofemoral pain is a clinical diagnosis, characterized by non-traumatic onset and diffuse anterior knee pain that is aggravated by knee-loading activities.^4^ The most reported painful activities are squatting, stepping-up and down, and prolonged sitting and/or kneeling.^5^ Clinicians often use these pain-provoking activities during the examination and track them over a course of treatment.^6^^,^^7^^,^^8^^,^^9^ In clinical practice, functional status can be assessed using both self-reported outcome measures and performance-based evaluations. While self-reported measures capture an individual’s perceived limitations in the context of their condition, performance-based tests quantify these limitations through standardized tasks such as squatting, stair ascent/descent, and single-leg hops.^10^^,^^11^

Despite the common use of these clinical functional tests, the reporting guide for patellofemoral pain (REPORT-PFP)^12^ does not include these condition defining activities. The REPORT-PFP recommends reporting a measure of pain severity and a condition-specific patient reported outcome–suggesting the Anterior Knee Pain Scale (Kujala Patellofemoral Scale)^13^ and the Knee Injury and Osteoarthritis Score Patellofemoral subscale (KOOS-PF)^14^ for the latter. This discrepancy highlights an important gap: although tests such as squatting and step-up/down are widely employed to assess functional performance in individuals with PFP, their relationship with validated PROMs remains poorly understood. Only a few studies have explored the association between clinical functional tests and PROMs in this population. For example, Nunes et al.^15^ investigated the relationship between stair climbing, chair stand, step-down, forward and side hop tests with Kujala and KOOS-PF scores, and found that only stair climbing time was moderately correlated with lower Kujala scores. Similarly, Zamboti et al.^9^ found significant association only between the 30-seconds sit-to-stand test and AKPS, despite evaluating other tests, including sitting-rising, stair-climb, stair-descent, and the six-minute step tests.

Function-based assessments offer valuable, low-cost, and time-efficient means to complement patient-reported data, providing a more comprehensive understanding of functional impairment.^6^^,^^10^ Understanding whether these commonly used clinical functional tests relate to patient-reported improvements would help clinicians monitor progress and adapt interventions more effectively throughout the treatment. Therefore, the aim of this study was to investigate how changes in clinical functional tests of step-up, step-down and squat are associated with changes in patient-reported outcomes among individuals with PFP.

## Methods

### Study design

This was a secondary analysis of data from a previously published randomised controlled trial that compared 12-week outcomes in individuals with PFP who had received either foot orthoses or underwent a 4-week hip exercise program.^7^^,^^16^

### Participants

Volunteers were recruited from Brisbane (Australia) and Aalborg (Denmark). The trial was prospectively registered (ACTRN12614000260628) and adhered to the principles of the Declaration of Helsinki.^17^ Ethical approval was granted by the University of Queensland Medical Research Ethics Committee (2013000981) and the Ethics Committee in the North Denmark Region (N-20140022). The participants provided informed consent prior to participation.

In the original trial, volunteers were screened for the following eligibility criteria: age between 18–40 years, presence of anterior, retro or peri‑patellar pain of insidious onset, exacerbated by at least two activities that load the patellofemoral joint (stepping-up and down, squatting, jogging/running, prolonged sitting), self-reported pain of at least 3/10 on a numerical pain rating scale (0 indicating no pain, and 10 indicating the worst imaginable pain) over the previous week, and a minimum duration of 6 weeks of symptom persistence. Additional criteria involved tenderness on palpation of the patellar borders; reproduction of symptoms completing a step-down (25 cm step) or double leg squat. Exclusion criteria for the original trial were reported traumatic onset of symptoms, concurrent injuries or pathologies affecting other knee structures that manifested with similar symptoms, a history of significant knee or other lower limb surgery, patellofemoral dislocation or subluxation, Osgood-Schlatter’s disease, Sinding-Larsen-Johansson syndrome, positive patellar apprehension test, or evidence of knee joint effusion. Volunteers were also excluded if they presented with any foot condition that prevented the use of foot orthoses, pain originating from the hip, pelvis or lumbar spine, current use of anti-inflammatory or corticosteroid medication (including injections) or had undergone prior treatment for PFP or other conditions that involved hip exercises or foot orthoses.^7^^,^^18^^,^^19^

The current study included an additional exclusion criteria: volunteers were excluded from the analysis if they reached 25 repetitions at both the initial and follow-up assessments in any of the proposed clinical functional tests. This was to remove the influence of ceiling effects from the data analysis.

### Assessment protocol

Participants were assessed at baseline and 12 weeks after treatment. Data from the affected limb was used for analysis and in cases with bilateral symptoms, the most symptomatic knee was used. Two assessors were responsible for collecting data in Aalborg (CS and MB) and one (MM) in Brisbane. All were physiotherapists with extensive clinical and research experience that attended training sessions provided by lead investigators (BV, MSR and MM).

### Clinical functional tests

Participants performed the 3 clinical tests of function in a fixed order of step-up, step-down and squat. The step-up and step-down tests were performed on a single 25 cm step at a rate of 96 beats per minute (i.e., participant had to step up/down on each beat). The squats were performed with feet shoulder width apart, squatting down until the participant was able to touch both lateral malleoli with their fingers. They did this at a rate of two beats down and two beats up, to a metronome set to 96 beats per minute. The test end points were either at the first onset of symptoms, at first increase in any existing symptoms, or when the participant reached a maximum of 25 repetitions without onset of pain.^7^ These in-clinic functional tests have been used in previous clinical trials as they are commonly reported as aggravating activities by patients with PFP.^18^, ^19^, ^20^

### Outcome measures

The patient reported outcome measures were the Kujala Patellofemoral Scale, the Knee Injury and Osteoarthritis Outcome Scale (KOOS), and the Global Rating of Change (GROC).

The KOOS is a questionnaire containing 5 separate subscales (symptoms, pain, activities of daily living function, sporting and recreation function and quality of life) that assess the patient’s perception of their knee and symptoms. Each subscale consists of standardized answers (five Likert scales) with a score of 0–4 for each question. Each subscale was normalized to a scale of 0–100 (0 representing extreme problems, and 100 representing no problems).^7^^,^^21^ A change of 8–10 points is suggested to represent a clinically significant change in symptoms.^21^ This questionnaire has good evidence for reliability and responsiveness to allow assessment of patient’s change over time.^21^^,^^22^

The Kujala Patellofemoral Scale is a 13-item questionnaire assessing multiple knee functions activities under varying loads, and the respective responses that best represented the participant’s symptoms.^7^^,^^23^ Each item is weighted separately and then summed overall, with score range from 0 (total incapacity) to 100 points (pain free full function).^7^^,^^23^ A change of 10 points is considered as the minimum clinically important difference in patients with PFP.^23^ This questionnaire is recommended for being reliable, valid, responsive and sensitive to change in patient’s symptoms.^23^, ^24^, ^25^

The GROC scale is a 7-point Likert scale with a range of response options that represent improvement through to worsening of the condition. At 12-weeks the participant rated their perception of any change in their symptoms since baseline.^26^ Participants were asked: “How would you describe your knee pain now, compared to before you began the treatment?” They selected the category best representing their change from the following options: *much better, better, a little better, no change, a little worse, worse, and much worse*.^7^^,^^26^ Global rating of change scales have been shown to be sensitive for measuring patient’s improvements in previous studies investigating treatments.^26^, ^27^, ^28^

### Statistical analysis

Descriptive statistics were calculated, and scatter plots were used to explore data distribution. Continuous variables were expressed with mean and standard deviation. We calculated the change scores (difference in scores between the follow-up visit at 12-weeks and baseline) for all the clinical functional tests for the affected limb (squat, step-up and step-down) and all outcome measures (Kujala and KOOS) except for GROC. The GROC was only measured at the 12-week follow-up visit. For analysis, GROC responses were dichotomized and the responses “much better” and “better” represented successful outcome.^29^

We performed a multivariate regression analysis to determine whether the independent variables (change scores in the clinical functional tests), significantly predict the results of dependent variables (change scores in Kujala and KOOS) when considered simultaneously. Regression assumptions were tested before analysis. Linearity between predictors and outcome variables was assessed via scatterplots and residual plots; independence of errors was checked using the Durbin-Watson statistic; homoscedasticity was assessed by visual inspection of residuals; normality of residuals was tested with Q-Q plots and the Shapiro-Wilk test; and multicollinearity was assessed using variance inflation factors (VIFs), with values below 2.0 for all predictors. The model was adjusted using Type III sum of squares. To evaluate the overall effect of the independent variables on the set of dependent variables, multivariate tests (Wilks’ Lambda) were conducted to investigate whether the independent variables, collectively, had a statistically significant association with the dependent variables. In addition to the global significance, univariate tests were performed to assess the individual effects of each independent variable on each dependent variable separately. Regression coefficients (β) were examined for each association, indicating the direction and magnitude of the effects. To quantify the practical importance of the results, effect sizes were calculated using Partial Eta Squared, which provides a measure of the proportion of variance in each dependent variable explained by each independent variable, controlling for the others. A binary logistic regression was performed using the forward conditional selection method to investigate whether the change in the number of pain-free repetitions in clinical functional tests was associated to the dichotomized perceived improvement after treatment (GROC). The inclusion and exclusion criteria for the variables were defined as *p* ≤ 0.05 and *p* ≥ 0.10, respectively. Model fit was evaluated using the Hosmer and Lemeshow test, while the explained variance was assessed using Nagelkerke's R². The predictive significance of the variables was determined through the Wald test, and their impact was expressed as odds ratios (Exp(B)) with 95 % confidence intervals. All statistical analyses were performed using SPSS software (SPSS Inc., Chicago, IL, USA), statistical significance was determined using an *α* level of 0.05.

## Results

**Fig. 1:**
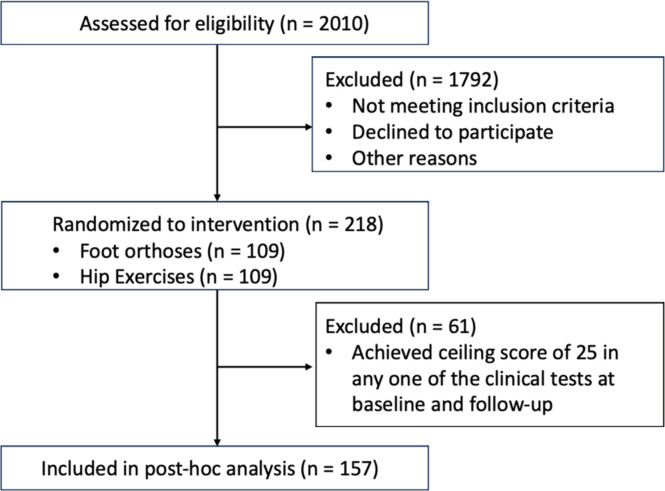
Participant flow chart showing exclusions for this secondary analysis (adapted from the original RCT^16^).

Of the 218 participants with data available, 61 were excluded because participants achieved a maximum of 25 repetitions on step-up, step-down or squat tests at both the baseline and 12-week follow-up (Fig. 1), without onset or increase in symptoms, representing a ceiling effect. The number of participants who reached 25 repetitions for each test was as follows: only step-up: 24; only step-down: 3; only squat: 3; both step-up and down: 18; both step-up and squat: 6; and all three tests: 7 participants.

The remaining 157 participants were included in the data analysis. Descriptive baseline data characteristics for the entire sample are summarized in Table 1. In addition, Table 2 summarizes data from the follow-up measurement, mean difference and the standardized response mean.

**Table 1:** Baseline characteristics of the 157 participants included in this analysis and the 61 who were excluded because they reached the maximum step-up count of 25 repetitions. Data expressed as mean (SD) unless otherwise specified.

Participant characteristics	Included in the analysis (*n* = 157)	Excluded from the analysis (*n* = 61)
Sex, n female (%)	113 (72 %)	38 (62.3 %)
Bilateral symptoms, n (%)	119 (75.8 %)	27 (44.3 %)
Most problematic knee, n right (%)	68 (43.3 %)	43 (70.5 %)
Age, years	27.5 (6.0)	27.8 (6.0)
Height, cm	170.5 (9.7)	173.1 (9.1)
Mass, kg	74.3 (17.2)	73.0 (14.7)
BMI, kg/m2	25.5 (5.2)	24.2 (3.8)
Duration of symptoms, months	60.8 (66.0)	36.0 (42.5)

Abbreviation list: n, number; BMI, body mass index; KOOS, Knee Injury and Osteoarthritis Outcome Scale.

**Table 2:** Baseline and follow-up, mean difference and the standardized response mean data.

Variable	Baseline mean (SD)	Follow-up mean (SD)	Mean difference (95 %CI)	Standardized Response Mean (SRM) (95 %CI)
Kujala Patellofemoral Scale /100	69.8 (9.6)	78.1 (11.3)	8.3 (6.1; 10.4)	0.7 (0.5;0.9)
KOOS Symptoms /100	67.3 (14.7)	73.9 (15.8)	6.6 (4.4; 8.7)	0.5 (0.4; 0.7)
KOOS Pain /100	68.1 (11.7)	77.6 (13.9)	9.5 (6.8; 12.2)	0.6 (0.4; 0.8)
KOOS Activities of Daily Living /100	78.0 (12.7)	85.3 (13.4)	7.3 (4.6; 10.0)	0.5 (0.3; 0.7)
KOOS Sporting and Recreation /100	49.9 (21.4)	68.8 (20.9)	18.9 (14.2; 23.7)	0.7 (0.5; 09)
KOOS Quality of Life /100	44.8 (16.8)	57.4 (18.2)	12.7 (8.9; 16.5)	0.6 (0.4; 0.7)
Step-up, n	11.7 (7.3)	16.6 (7.9)	5.0 (3.0; 6.9)	0.5 (0.3; 0.7)
Step-down, n	6.1 (5.4)	11.3 (8.5)	5.3 (3.4; 7.1)	0.5 (0.3; 0.7)
Squat, n	6.4 (4.9)	11.4 (8)	5.0 (3.4; 6.6)	0.6 (0.4; 0.8)

Abbreviation list: n, number; KOOS, Knee Injury and Osteoarthritis Outcome Scale.

Table 3, Table 4 show the results for the overall model significance and tests of between-subjects effects from the multivariate regression analysis, respectively. The multivariate tests showed that among the independent variables, step-up presented a significant effect (*F* = 2.515, *p* = 0.027), with a contribution of 13.8 % (Partial Eta Squared = 0.138) on the dependent variables. On the other hand, step-down (*F* = 1.566, *p* = 0.166) and squat (*F* = 1.147, *p* = 0.342) did not have significant effects on the dependent variables, with Partial Eta Squared values of 0.091 and 0.068, respectively. This indicates that these variables had a marginal impact on the dependent variables, explaining a smaller proportion of the variation.

**Table 3:** Multivariate results table (overall model significance).

Effect	Value	F	Hypothesis df	Error df	Sig.	Partial Eta Squared	Observed Power
Intercept	.778	4.473	6.000	94.000	.001	.222	.980
Step-up	.862	2.515	6.000	94.000	.027	.138	.817
Step-down	.909	1.566	6.000	94.000	.166	.091	.578
Squat	.932	1.147	6.000	94.000	.342	.068	.432

Results presented for Wilks' Lambda test.

**Table 4:** Outputs results for tests of between-subjects effects of the multivariate regression analysis and the binary logistic regression.

Dependent variables	Independent variables	F	Sig (p)	Partial Eta Squared	Adjusted R^2^	Coefficient (B)	Standard Error (SE)		T	Sig. (p)	95 %CI
**Kujala**											
	Corrected model	10.345	**<0.001**	.239	.216						
	Intercept	14.741	<0.001	.130		4.742	1.235		3.839	.000	2.291, 7.193
	Step-up	2.126	.148	.021		.204	.140		1.458	.148	-0.073, 0.481
	Step-down	4.159	**.044**	.040		.283	.139		2.039	.044	.008, 0.558
	Squat	1.650	.202	.016		.211	.164		1.284	.202	-0.115, 0.537
**KOOS Symptoms**											
	Corrected model	5.303	**.002**	.138	.112						
	Intercept	7.121	.009	.067		3.786	1.419		2.669	.009	.971, 6.601
	Step-up	5.497	**.021**	.053		.376	.160		2.345	.021	.058, 0.694
	Step-down	1.167	.283	.012		.172	.159		1.080	.283	-0.144, 0.488
	Squat	.106	.745	.001		-0.062	.189		-0.326	.745	-0.436, 0.313
**KOOS Pain**											
	Corrected model	7.406	**<0.001**	.183	.159						
	Intercept	9.900	.002	.091		5.418	1.722		3.146	.002	2.001, 8.834
	Step-up	5.765	**.018**	.055		.467	.195		2.401	.018	.081, 0.854
	Step-down	.717	.399	.007		.164	.193		.847	.399	-0.220, 0.547
	Squat	.402	.527	.004		.145	.229		.634	.527	-0.309, 0.600
**KOOS ADL**											
	Corrected model	8.516	**<0.001**	.205	.181						
	Intercept	3.534	.063	.034		3.222	1.714		1.880	.063	-0.179, 6.623
	Step-up	2.240	.138	.022		.290	.194		1.497	.138	-0.094, 0.675
	Step-down	3.666	.058	.036		.369	.192		1.915	.058	-0.013, 0.751
	Squat	.822	.367	.008		.207	.228		.906	.367	-0.246, 0.659
**KOOS Sport and Recreation**											
	Corrected model	15.278	**<0.001**	.316	.296						
	Intercept	22.733	.000	.187		12.124	2.543		4.768	.000	7.079, 17.170
	Step-up	12.335	**.001**	.111		1.010	.288		3.512	.001	.439, 1.580
	Step-down	4.873	**.030**	.047		.630	.286		2.208	.030	.064, 1.197
	Squat	.093	.762	.001		-0.103	.338		-0.304	.762	-0.774, 0.568
**KOOS QoL**											
	Corrected model	7.121	**<0.001**	.177	.153						
	Intercept	10.592	.002	.097		7.566	2.325		3.255	.002	2.953, 12.179
	Step-up	4.095	**.046**	.040		.532	.263		2.024	.046	.010, 1.054
	Step-down	.822	.367	.008		.237	.261		.907	.367	-0.281, 0.755
	Squat	.913	.342	.009		.296	.309		.956	.342	-0.318, 0.909
**Binary Logistic Regression**											
**GROC**											
[X^2^(1) = 13.494; *p* = .000, R^2^ Nagelkerke = 0.160]											
**Step 1**	**B**	**S. E**	**Wald**	**df**	**Exp(B)**	**95%CI for EXP (B)**	**Sig**	**Hosmer and Lemeshow Test: Chi-square (df)**			**Hosmer and Lemeshow Test Sig**
Step-up	.076	.022	11.430	1	1.079	1.032 – 1.128	.001	3.845 (7)			.797
Constant	-0.146	.227	.415	1	.864		.519				

Regression models were performed using the change scores (difference in scores between the follow-up visit at 12 weeks and baseline) for clinical tests and outcome measures, except for GROC. KOOS, Knee Injury and Osteoarthritis Outcome Scale; GROC, Global Rating of Change.

The between-subjects effects analysis examined how each independent variable contributed individually to the variation in the dependent variables. The proportion of variance explained by the multivariate regression models ranged from 11.2 % to 29.6 %, with the KOOS Sport and Recreation model showing the highest explanatory power (29.6 %), followed by Kujala (21.6 %), KOOS ADL (18.1 %), KOOS Pain (15.9 %), KOOS QoL (15.3 %), and KOOS Symptoms (11.2 %). Among the predictor variables, performance in the step-up test was the most consistent and significant contributor across models. It was significantly associated with KOOS subscales of Symptom (*p* = .021; 5.3 % of variance explained), Pain (*p* = .018; 5.5 %), Sport & Recreation (*p* = .001; 11.1 %) and Quality of Life (*p* = .046; 4.0 %). The step-down test showed significant effects on Kujala (*p* = .044; 4 %) and KOOS Sports & Recreation (*p* = .030; 4.7 %). The squat test did not show significant effects on any of the dependent variables (all *p* > 0.20). Both the multivariate analysis and the between-subjects effects analysis agree in identifying step-up as the most significant factor, with a stronger impact on the set of dependent variables.

For the binary logistic regression, only the step-up test showed a significant association (*p* = .001) between its change scores and the dichotomized GROC, explaining 16.0 % of the variance.

## Discussion

Out of 218 participants from a previous RCT comparing foot orthoses and hip exercises, we had complete data from 157 to include in this secondary analysis. We found that step-up test was associated with all PROMs, except KOOS ADL subscale and Kujala. The relationship was positive, meaning that an increase in step-up was associated with improvements in these PROMs. The squat test appears to be less informative and can’t be recommended based on the current data. Our findings suggest that the step-up test may be a useful in-clinic measure for tracking improvements in function.

Treatment with either hip exercises or a combination of foot orthoses and foot exercises improved all the outcomes reported herein. The association between the improvements in step tests and patient-reported outcomes could be explained in several ways. One possibility is that the treatment improved the participant’s neuromuscular system which may account for the observed improvements in clinical functional tests and patient-reported outcomes. A previous study also reported similar improvements in functional tests following a physical therapy intervention; which consisted of mainly neuromuscular exercises targeting the gluteal and quadricep muscles.^19^ The effect of the treatment on muscle capacity might account for these findings, as another study reported that reduced hip muscle strength is associated with lower functional performance (fewer step-down repetitions and shorter forward hop for distance).^15^ The pain reduction and improvements in physical function observed in our study may also be explained by neurophysiological mechanisms, such as exercise-induced hypoalgesia and the release of endogenous opioids.^30^

The finding that step-up and down tests similarly improved over the 12-weeks alongside the finding that only step up was associated with PROMs requires exploration. This stronger association with the step-up test may be because it places lower patellofemoral joint reaction force and stress compared to other tasks like the forward step down.^31^ Additionally, the clinical functional tests used are pain threshold tests, and participants were able to perform nearly twice the number of repetitions on step-up at baseline compared to the step-down and squat tests. This suggests that improvement in their capacity to perform the test was more substantial, given their already higher baseline performance. It is also possible that, due to increased load during step-down, participants felt higher pain intensities during this test, meaning that even with similar change scores, the improvement had a smaller effect on the PROMs. It is also important to note that most of the 61 participants excluded for reaching the maximum of 25 repetitions did so on the step-up test. Therefore, it is possible that their improvements in change scores may have been greater but were not captured in the analysis.

Interestingly, the change score for the squat test did not contribute to any of the models. One possible explanation could be linked to the nature of the movements. While the squat is an important functional movement, in questionnaires like the KOOS and Kujala, many questions are associated with activities more directly related to locomotion and daily functionality (e.g., walking, climbing stairs, carrying weight, or changing/moving positions), which may align more closely with the step-up and step-down movement than with the squat.

The models with clinical functional tests included in the analysis explained from 11 to 30 % of the variance, with generally weak to moderate association, consistent with the multifactorial nature of PFP and highlighting that changes in functional tests explain only part of the variance in patient-reported improvements. There are other variables not included in the analysis that play a role in explaining changes in the PROMs. For example, psychological factors, which we did not assess in this study, might influence changes in PROMs. As an example, kinesiophobia is associated with self-reported pain and disability in women with PFP, while patellofemoral joint loading variables are not.^32^ Other studies have shown a positive association between kinesiophobia, pain catastrophizing, anxiety, depression and fear of movement with pain, and a negative association with function, meaning that higher levels of these psychological traits are associated with increased pain and reduced function.^33^, ^34^, ^35^ Self-efficacy, in particular, has been identified as a strong predictor of successful outcomes for patients with persistent pain, irrespective of the intervention provided.^36^ Furthermore, higher self-efficacy is linked to better function and health-related quality of life.^37^ Thus, it is possible that in addition to improving the number of repetitions in the clinical functional tests, the treatment provided by the RCT also positively influenced the patient’s self-efficacy (their belief in their ability to perform an activity or overcome a challenge)^36^ and contributed to improved function in clinical tests and better self-reported outcomes. This is all purely speculative, as this is the first study testing the association between change scores in the pain threshold tests of squatting and stepping-up and down with PROMs in individuals with PFP.

Since functional test performance may be dependent on factors such as neuromuscular and biomechanical factors,^15^^,^^38^ future studies should explore whether changes in these domains are associated with improvements in PROMs. For example, King et al.,^39^ investigated lower-limb joint moment impulses during walking and their association with KOOS subscales in people with patellofemoral osteoarthritis. They reported that these associations varied depending on the anatomical plane, joint compartment involved, and disease severity, highlighting the complex relationship between joint mechanics and symptoms, that may inform treatment strategies. Furthermore, approximately 30 % of the data were excluded due to ceiling effects, suggesting that the functional tests employed — particularly the step-up— may not have been sufficiently challenging or demanding^38^ for individuals with higher baseline function. This limited sensitivity likely contributed to the weak to moderate associations observed, indicating that while the step-up may have limited discriminatory capacity at the higher end of functional performance, it retains clinical utility for detecting meaningful differences in patients with lower to moderate functional levels. In clinical practice, functional tests should avoid substantial ceiling effects (i.e., many participants achieving the highest possible scores) or floor effects (many scoring the lowest), and should demonstrate sensitivity to change and responsiveness.^40^ Future studies should consider more demanding and provocative functional assessments (i.e., single leg vertical and lateral hopping tests), and investigate what constitutes a meaningful change for commonly used tests.^40^ Additionally, test selection should be informed by the physical activity level of the population being assessed,^38^ to better reflect the heterogeneity in functional capacity among individuals with PFP and improve the detection of clinically relevant associations.

There are limitations to this study that should be considered. We stopped the tests after a maximum of 25 repetitions was reached without the onset of pain. This decision aimed to standardize data collection by limiting the duration of the testing and avoiding exacerbating the symptoms. To mitigate this ceiling effect, we excluded participants who achieved 25 repetitions at both the baseline and 12-week follow-up. This exclusion was necessary because when a significant proportion of participants reaches the maximum possible score (ceiling), the clinical test of function may not effectively measure change in performance.^41^ We did not measure knee flexion angle and there is a possibility that the amount of knee flexion achieved by each individual participant, and thus the joint load and possibly pain onset, would be different between participants. We suggest that this does not impact on our findings because we analysed the change in numbers of repetitions of steps/squats, which would mitigate inter-subject variability in this regard. Whilst the potential impact of test order should be acknowledged, as pain and fatigue could affect performance, given the step-up test was first performed, we proposed test-order influence to be minimal. This study involved an exploratory secondary analysis of data from a RCT. While the sample size was determined based on the primary RCT objectives, we propose that the number of participants was adequate for the regression models, which included only three independent variables per model.

## Conclusion

Improvements in the clinical functional tests of step-up and step-down, but not in the squat test, were associated with improvements in PROMs. The step-up test showed the strongest association. Clinicians using these tests as in-clinic are likely to expect changes reflected in PROMs. This reinforces the clinical value of combining in-clinic functional tests with PROMs to monitor and guide patient’s progress throughout rehabilitation.

## Declaration of competing interest

The authors declare no competing interest.

## Funding

This research did not receive any specific grant from funding agencies in the public, commercial, or not-for-profit sectors.

## Ethical approval

We confirm that ethical approval was granted by the University of Queensland Medical Research Ethics Committee (2013000981) and by the Ethics Comittee in the North Denmark Region (N-20140022), and the participants gave informed consent to the work.
